# Mcl-1 Is Up Regulated by Prenylated Coumarin, Umbelliprenin in Jurkat Cells

**Published:** 2014

**Authors:** Omid Gholami, Mahmood Jeddi-Tehrani, Mehrdad Iranshahi, Amir Hassan Zarnani, Seyed Ali Ziai

**Affiliations:** a *Physiology and Pharmacology Department, Faculty of Medicine, Sabzevar University of Medical Sciences.*; b *Monoclonal Antibody Research Center, Avicenna Research Institute, ACECR, Tehran, Iran. *; c *Biotechnology Research Center and School of Pharmacy, Mashhad University of Medical Sciences, Mashhad, Iran.*; d *Nanobiotechnology Research Center, Avicenna Research Institute, ACECR, Tehran, Iran.*; e *Pharmacology Department, Faculty of Medicine, Shahid Beheshti University of Medical Sciences, Tehran, Iran.*

**Keywords:** Umbellprenin, Jurkat cell, Apoptosis, Mcl-1, Real time PCR

## Abstract

Chronic lymphocytic leukaemia (CLL) is the most common B-cell malignancy in the western world and exists as subtypes with very different clinical courses. Myeloid cell leukemia 1 (Mcl-1) is one member of Bcl-2 family proteins that has been shown to be expressed in various tissues and malignant cells, including CLL, where its expression is significantly associated with a failure to achieve complete remission following cytotoxic therapy.

Induction of apoptosis by prenylated coumarin, umbelliprenin, in Jurkat cells was previously shown. We examined whether umbelliprenin can down-regulate Mcl-1 gene and protein in Jurkat cells. In this regard cells were incubated by umbelliprenin, and then down- regulation of Mcl-1 gene was studied by Real Time PCR method. Moreover, down-regulation of Mcl-1 protein was studied by western blot analysis.

We showed that, expression of Mcl-1 mRNA was increased from 1 hour to 3 hours incubation٫ but this increase has a scale down pattern. Moreover umbelliprenin could inhibit Mcl-1 protein. In conclusion umbelliprenin treatment modulates Mcl-1 expression at both the transcriptional and posttranslational levels.

## Introduction

Chronic lymphocytic leukemia (CLL) is a type of leukemia that is characterized by accumulation of mature lymphocytes with typical B-cell markers. Leukemic lymphocytes accumulate both in the bone marrow and the peripheral blood. This accumulation is due to intrinsic defects in their apoptotic machinery and/or dysregulated production of survival signals from their microenvironment. There is a balance between the anti- and pro-apoptotic proteins and this maintains the rheostat of B cells and all hematopoietic cells. Myeloid cell leukemia 1 (Mcl-1) is one of the members of anti-apoptotic Bcl-2 family proteins. This protein surfaces as the most significant anti-apoptotic protein associated with normal as well as malignant B lymphocytes (1).

Mcl-1 is an essential protein during lymphoid development and maintenance of mature T and B lymphocytes (2), and the expression level of anti-apoptotic proteins in normal and malignant lymphocytes is in concordance with its role in survival (3). It has been shown that high levels of Mcl-1 and Bcl-2 mRNA and protein were founded in CLL, which are inversely correlated with *in-vitro* response to chemotherapeutic agents and with the failure of CLL patients to respond to fludarabine therapy (4). Conversely, down-regulation of Mcl-1 protein expression by antisense oligonucleotides or through indirect Mcl-1 transcription and translation inhibitors results in cell death during *in-vitro* culture or *in-vivo* therapy. In addition, over expression of Mcl-1 prolongs the survival of CLL cells exposed to a variety of apoptosis-inducing stimuli (3). These key pieces of evidence establish Mcl-1 as a critical survival factor for CLL.

**Figure 1:**
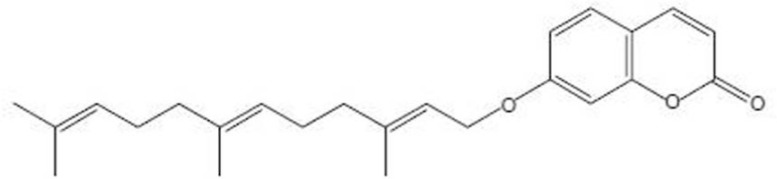
umbelliprenin.

Umbelliprenin (Figure 1), a sesquiterpene coumarin, is synthesized by various *Ferula* species. *Ferula* is a genus of about 170 species of flowering plants in the family Apiaceae, native to the Mediterranean region east to central Asia (5). The genus *Ferula (Apiaceae)*, contain both useful (*Ferula asafoetida L., Ferula gummosa Boiss. *and *Ferula hermonis Boiss*.) and toxic (*Ferula communis L*.) plants. The use of plants from the *Ferula* genus (Family *Umbelliferae*) is documented in the traditional medicine of the Mediterranean region since the Greek and Roman times. 

Umbelliprenin has been also found in various plant species consumed as food or used for food preparation such as celery, *Angelica archangelic*, *Coriandrum sativum*, and *Citrus limon*. Umbelliprenin has been reported to inhibit growth of some pathogenic bacterial strains (6) and to prevent red pigment production in *Serratia marcescens* (7). It has also been reported to inhibit matrix metalloproteinases activity (8), possess anticoagulant, antileishmanial against promastigotes (6, 9), and antiproliferative activity (-). 

Given the ability of umbelliprenin to induce apoptosis in jurkat cells, we investigated the efficacy of umbelliprenin to down-regulate Mcl-1 gene and protein. 

## Experimental

*Plant material and umbelliprenin isolation*

Umbelliprenin (C_24_H_30_O_3_, MW: 366) was purified (>95%) as previously described (9) from dried roots of *Ferula szowitsiana* D.C collected from the mountains of Golestan forest (Golestan province, Iran). A voucher specimen of the roots (no. M1001) has been deposited at the Department of Pharmacognosy and Biotechnology, Faculty of Pharmacy, Mashhad University of Medical Sciences. For this study, umbelliprenin was diluted in DMSO. Immediately before use, it was diluted in the culture medium to obtain a final DMSO concentration of 0.5% (v/v).

*Cell culture*

Jurkat cells were prepared from National Cell Bank of Iran (Pasteur institute, Tehran, Iran). Cells were grown in RPMI 1640 culture medium containing 10% fetal bovine serum (FBS), Penicillin (10,000 U/mL) and Streptomycin (10 mg/mL) in several culture flasks in a CO_2_ (5%) incubator at 37 °C and 95% humidity, until totally 50×10^6^ cells. Cells were then frozen in FBS containing 10% dimethyl sulfoxide (DMSO) and stored in liquid nitrogen (5×10^6^ cells/vial). The viability of cryopreserved cells was determined by trypan blue staining immediately upon thawing. Only cells whose viability exceeded 93% (range, 93.4%-99%) were used in this study.

*Western blot analysis*

Jurkat cells were incubated by umbelliprenin (50 µM) in 37 ºC and 5% CO_2_ for 3, 6 and 16 hours. After that cells were collected and lysed with the lysis buffer (EDTA 0.5 M 1 mL, Tris–HCl pH 7.4, 50 mL, NaCl 0.88 g, NaF 0.0042 g, Na_4 _P_2 _O_7 _0.89 g, SDS 0.1 g, Triton 1 mL, glycerol 1 ml, protease inhibitor cocktail I (1X; Roche), phosphatase inhibitor cocktail II (1X; Sigma)). Protein concentration was determined using the Bradford method (13). Cell lysates containing 20 µg of total protein were loaded onto 12% SDS–polyacrylamide gels with Tris/glycine running buffer and transferred to polyvinylidene difluoride (PVDF) membranes (Roche USA). Membrane was blocked with blocking buffer (5% skim milk, NaCl 8.7 g, Tris–Base 6.05 g and D.D.W. to 1000 mL pH 7.4) for 1 h at room temperature and incubated with the primary antibody (anti-Mcl-1 Rabbit Ab. 1:1000 (Cell Signaling), diluted in 5% skim milk) at 4 °C overnight. After washing with Tris–buffered saline containing 0.1% Tween-20, the membrane was incubated with an Anti-rabbit IgG antibody conjugated with horseradish peroxidase (1:3000, (Cell Signaling), diluted in 5% skim milk, NaCl 8.7 g, Tris–Base 6.05 g and D.D.W. to 1000 ml pH 7.4) at room temperature for 1 h. The blots were incubated with antibodies that recognize β-actin (mouse mAb, Avicenna Research Institute, Tehran, Iran) as loading control. The signal was detected using an enhanced chemiluminescence Western blotting detection system (Amersham Bioscience). 

*Real Time-PCR analysis*

Jurkat cells were incubated by umbelliprenin (50 µM) in 37 ºC and 5% CO_2_ for 1, 2 and 3 hours. Total cellular RNA was isolated. The quantity and quality of the total RNA was verified with the PicoDrop spectrophotometer (alpha biotech, Cambridge, UK) according to the manufacturer’s instructions. Complementary DNA was synthesized from 2 µg total RNA using the “cDNA Synthesis for RT-PCR Protocol, National Institutes of Health” (14). Polymerase chain reaction (PCR) was performed in 4 replicates in 20 µL reaction volumes using 1 µL cDNA, 10 µL SYBR Green master mix (Primer Design, Precision 2X qPCR Master Mix), and 0.4 µL of each primer. PCR was performed using Mcl-1 primers (5′-CCA AGA AAG CTG CAT CGA ACC AT-3′ and 5′-CAG CAC ATT CCT GAT GCC ACC T-3′) and β-actin primers (5′_AGC CTC GCC TTT GCC GA-3′ and 5′_CTG GTG CCT GGG GCG-3′). Samples were amplified in a 7500 Real Time PCR System (Applied Biosystems, Foster City, CA) for 50 cycles using the following PCR parameters: 95 °C for 10 minutes, 95 °C for 15 seconds, and 60 °C for 1 minute. Gene expression was quantitated using the comparative CT method of relative quantification using 7500 System SDS software (Applied Biosystems). Finally data were analyzed by Rest-rg software.

*Statistical analysis*

One way ANOVA test was used for statistical analysis. The p-value was considered significant when it was less than 0.05.

## Results

*Umbelliprenin down-regulates Mcl-1 protein*

Mcl-1 is a prosurvival member of the Bcl-2 family proteins whose expression levels in leukemic cells are associated with response to treatment both *in-vitro* and *in-vivo*. Previous studies in tissue culture have shown that persistent Mcl-1 expression is strongly associated with resistance to chlorambucil and fludarabine (4). Clinical studies have demonstrated that low Mcl-1 levels are found in patients who achieve complete remission after treatment (15). The level of Mcl-1 expression was found to be the best measure of clinical response in CLL patients. Thus, we examined whether umbelliprenin could modulate the level of Mcl-1 in jurkat cells *in-vitro*. Cells were incubated with umbelliprenin for 3, 6 and 16 hours and examined for changes in Mcl-1 expression by Western blot analysis. 

**Figure 2:**
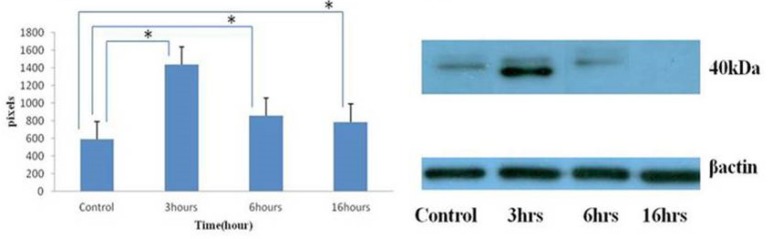
Changing in Mcl-1 protein expression by umbelliprenin (50 µM) on jurkat cells after 3, 6, 16 hours incubation. Umbelliprenin decreases Mcl-1 contents. β actin was used as a loading control. The ratio of each protein to β actin was calculated each time and showed as a column chart. Data are shown as mean ± standard deviation. *P<0.05.

A modest increase in Mcl-1 protein was observed after 3 hours following umbelliprenin exposure, followed by a decrease in Mcl-1 protein within 6 hours that continued through 16 hours. The expression of Mcl-1 protein following umbelliprenin exposure appeared to be biphasic demonstrating a transient increase in Mcl-1 protein during the initial stages of an apoptotic response. Down-regulation of full length Mcl-1 protein was likely due to posttranslational regulation by caspases (Figure 2).

*Umbelliprenin up-regulates Mcl-1 mRNA*

**Figure 3:**
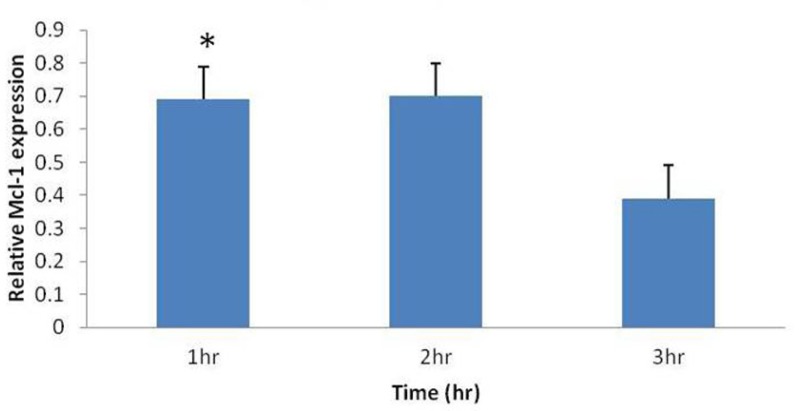
Increase in relative Mcl-1 mRNA expression (Mcl-1 expression _Treat_/ Mcl-1 expression _Control_), by umbelliprenin (50 µM) on jurkat cells after 1, 2, 3 hours incubation. Mcl-1 levels were normalized to β actin. Only after 1 hour incubation this increase was significant (*P= 0.026).

Some studies have demonstrated that Mcl-1 mRNA is up-regulated as part of an initial rapid cellular response to cytotoxic stimuli such as chemotherapeutic agents, calcium ionophores, pneumococcal infection and UV irradiation. To determine whether umbelliprenin could similarly affect Mcl-1 gene expression in jurkat cells, we examined Mcl-1 mRNA levels. Real-time PCR was performed on treated jurkat cells for 1, 2 and 3 hours, and then data were analyzed by Rest-rg software. This software calculates relative Mcl-1 expression (Mcl-1 expression _Treat_/ Mcl-1 expression _Control_), and shows this relative expression as a unique column (Figure 3). In each case, Mcl-1 mRNA increased in response to umbelliprenin relative to control specimens (Figure 3). This increase is significant for 1 hour treatment. For 2 and 3 hours treatment, Mcl-1 mRNA increased but this increase is not significant. Thus, umbelliprenin falls into the category of apoptosis-inducing agents that initially up-regulates Mcl-1 mRNA. 

Altogether, these data (alterations in Mcl-1 mRNA and protein) indicate that umbelliprenin treatment modulates Mcl-1 expression at both the transcriptional and posttranslational levels.

## Discussion

CLL is a disease characterized by the accumulation of apoptotic resistant lymphocytic cells. The natural product umbelliprenin was reported to induce apoptosis in jurkat T-CLL cells (11, 12). Therefore, we investigated whether exposure of jurkat cells to umbelliprenin could modulate the expression of the important Bcl-2 family member, Mcl-1, which is a molecular change associated with positive clinical outcome.

Mcl-1 is a member of the Bcl-2 gene family. Alterations in the balance of the corresponding proteins are commonly found in hematologic malignancies. Pepper et al. showed that a high ratio of Bcl-2/Bax expression and high expression of Mcl-1 are contributed to the pathogenesis of CLL (4, 16). Association between high Mcl-1 expression and the inability to achieve complete remission has been reported. This finding indicates that higher Mcl-1 protein expression is an indicator of adverse outcome for B-CLL patients (15). In another study, Mcl-1 was the only protein among a panel of antiapoptotic proteins found to be associated with chemoresistance *in-vitro* and the failure to achieve complete response in B-CLL patients (4). Conversely, patients who achieved complete remission exhibited low Mcl-1 levels, and *in-vitro* exposure to chemotherapeutic agents caused a reduction in its relative levels.

 Thus, it was important to perform *in-vitro* studies as a first step toward determining whether umbelliprenin could affect Mcl-1 expression in jurkat cells. We found that Mcl-1 levels were reduced in umbelliprenin-treated jurkat cells, and that Mcl-1 undergoes a complex multistep regulatory process. Down regulation of Mcl-1 presumably alters the balance of proapoptotic and antiapoptotic proteins and would facilitate mitochondrial activation of programmed cell death. Mcl-1 expression is emerging as a prognostic determinant of outcome and response(17). Our finding that umbelliprenin overcomes the apoptosis resistance present in CLL cells at least in part by down regulation of Mcl-1 bears potential significant clinical relevance. 
